# Electrophysiological characteristics of pressure overload-induced cardiac hypertrophy and its influence on ventricular arrhythmias

**DOI:** 10.1371/journal.pone.0183671

**Published:** 2017-09-01

**Authors:** Xiaowei Chen, Mu Qin, Weifeng Jiang, Yu Zhang, Xu Liu

**Affiliations:** Department of Cardiology, Shanghai Chest Hospital, Shanghai Jiao Tong University, Shanghai, China; University of Minnesota, UNITED STATES

## Abstract

**Objective:**

To explore the cardiac electrophysiological characteristics of cardiac hypertrophy and its influence on the occurrence of ventricular tachyarrhythmias.

**Methods:**

Adult C57BL6 mice were randomly divided into a surgery group and a control group. Thoracic aortic constriction was performed on mice in the surgery group, and cardiac anatomical and ultrasonic evaluations were performed to confirm the success of the cardiac hypertrophy model 4 weeks after the operation. Using the Langendorff method of isolated heart perfusion, monophasic action potentials (MAPs) and the effective refractory period (ERP) at different parts of the heart (including the epi- and endo-myocardium of the left and right ventricles) were measured, and the induction rate of ventricular tachyarrhythmias was observed under programmed electrical stimulus (PES) and burst stimulus. Whole-cell patch-clamp was used to obtain the I-V characteristics of voltage-gated potassium channels in cardiomyocytes of different parts of the heart (including the epi- and endo-myocardium of the left and right ventricles) as well as the channels’ properties of steady-state inactivation and recovery from inactivation.

**Results:**

The ratio of heart weight to body weight and the ratio of left ventricular weight to body weight in the surgery group were significantly higher than those in the control group (P < 0.05). Ultrasonic evaluation revealed that both interventricular septal diameter (IVSD) and left ventricle posterior wall diameter (LVPWD) in the surgery group were significantly larger than those in the control group (P < 0.05). Under PES and burst stimuli, the induction rates of arrhythmias in the surgery group significantly increased, reaching 41.2% and 23.5%, respectively. Both the QT interval and action potential duration (APD) in the surgery group were significantly longer than in the control group (P<0.01), and the changes showed obvious spatial heterogeneity. Whole-cell patch-clamp recordings demonstrated that the surgery group had significantly lower potassium current densities (I_Peak_, I_to_, I_Kur_, I_ss_, and I_K1_) at different parts of the heart than the control group (P < 0.01), and there were significant differences in the half-inactivation voltage (V_1/2_) and the inactivation-recovery time constant (τ) of I_to_ and I_Kur_ at different parts of the heart (P < 0.01) between the surgery group and the control group. In addition, the surgery group had significantly lower densities of I_Peak_, I_to_, and I_Kur_ in cells of the endo-myocardium (P < 0.05), and the changes showed obvious spatial heterogeneity.

**Conclusion:**

Changes in the current density and function of potassium channels contributed to irregular repolarization in cardiac hypertrophy, and the spatially heterogeneous changes of the channels may increase the occurrence of ventricular arrhythmias that accompany cardiac hypertrophy.

## Introduction

Cardiac hypertrophy, which is regarded as an adaptive process under the influence of continuous haemodynamic overload, is a common complication of hypertension, ischemic cardiomyopathy, valvular diseases, and many other cardiovascular diseases [1]. Without early intervention, cardiac hypertrophy leads to cardiac enlargement, congestive heart failure, and malignant arrhythmias, which are very common complications of cardiac hypertrophy, ultimately resulting in sudden cardiac death [2]. Although great progress has been achieved regarding the use of medication to treat this disease, its morbidity remains high at approximately 43% among people over 70 and more than 50% among severe hypertension patients [3]. Therefore, research has focused on the pathogenesis of ventricular arrhythmias, which occur during the occurrence and progression of cardiac hypertrophy, to identify effective control measures. Electrophysiological changes (electrophysiological remodelling) during the occurrence and progression of cardiac hypertrophy primarily manifest as repolarization delays, including extensions of the QT interval and APD, and a resulting increase in the dispersion of repolarization [4–6]. A previous study revealed that APD prolongation is attributable to alterations in multiple potassium channels [7] and that potassium channels that function in key ion transport during the repolarization phase in cardiomyocytes played a critical role in the occurrence of arrhythmias in a model of cardiac hypertrophy [8]. Studies have revealed that potassium current densities are decreased in patients with left ventricular hypertrophy [9]. It is not clear whether the heterogeneous distribution of channels is associated with the occurrence of arrhythmias in cardiac hypertrophy. With the establishment of a cardiac hypertrophy model, changes in the potassium-ion channel density and kinetics in different regions of the heart were detected in this study, and the results may provide a theoretical basis for the pathogenesis of arrhythmias that accompany cardiac hypertrophy.

## Materials and methods

All protocols conformed to the Guide for the Care and Use of Laboratory Animals published by US National Institutes of Health (NIH Publication No. 85–23, revised 1996) and were approved by The Institutional Animal Care and Use Committee at Shanghai Chest Hospital, Shanghai Jiao Tong University.

### 1. Animal model

All C57BL/6 mice for research (male, 10–12 weeks old) were provided by the animal experimental centre. The animals were reared in the room at constant temperature and humidity. All mice were anaesthetized with 3% pentobarbital sodium (50 mg/kg). The cardiac hypertrophy model was established by performing thoracic aortic constriction on the mice in the surgery group. Thoracotomy was performed on animals in the control group (sham group), but no treatment was performed on their aortas. A cardiac ultrasonic evaluation was carried out to confirm the success of the model 12 days after the operation.

### 2. ECG recording

Three percent pentobarbital sodium (prepared with normal saline) was administered to mice through an intraperitoneal injection at a dose of 0.03 ml/g. After 10–20 min, mice were placed on a constant-temperature heating plate (37°C). Ag/AgCl electrodes (Linton Instruments, Harvard Apparatus) were placed in the subcutaneous tissue of the four limbs and connected to an ECG amplifier (AD Instruments, Australia). The low-pass filter and high-pass filter were adjusted to 0.05 Hz and 1 kHz, respectively. After stabilization for 5 min, lead II ECG recording from the limbs was performed for 30 min.

### 3. Isolated heart preparation

A total of 100 IU of heparin sodium was administered to mice through intravenous and intraperitoneal injections to inhibit coagulation. Ten minutes later, the mice were killed, and their hearts were removed immediately and placed into 100% oxygen-saturated normal saline (4°C) to remove the pericardium and surrounding adipose tissue. A 22-gauge needle was inserted through the aorta under a microscope in a retrograde manner and then connected to a Langendorff heart perfusion apparatus (AD Instruments, Australia). Retrograde trans-aortic heart perfusion with 100% oxygen-saturated Tyrode’s-HEPES solution (mM: NaCl 130; KCl 5.4; CaCl_2_ 1.8; MgCl_2_ 1; Na_2_HPO_4_ 0.3; HEPES 10; glucose 10; the pH value was adjusted to 7.4 by adding NaOH; at a constant temperature of 37°C) was carried out at 2–2.5 ml/min, and the perfusion pressure was kept within the range of 60~80 mmHg. The entire procedure from removing the heart to achieving perfusion and heart resuscitation must be completed within 5 min, and recording should be started after stabilization for 20 min. Samples that were found with obvious ischaemia or arrhythmias within 20 min after perfusion were excluded.

### 4. MAP and bipolar ECG recording

A pair of platinum stimulating electrodes (at an interval of 1 mm) was placed on the outer myocardium at the right ventricular base for cardiac pacing. The frequency was 8 Hz, pulse width was 1 ms, and the stimulating voltage was 2 times the diastolic pacing threshold. MAP electrodes (Linton Instruments, Harvard Apparatus) were used to record the MAP of left and right ventricles. By using silver chloride bipolar electrodes (Linton Instruments, Harvard Apparatus), which were 0.25 mm in diameter and coated with Teflon, a bipolar ECG recording was carried out to record the waveforms of ventricular arrhythmias. All signals recorded using a MAP amplifier (AD Instruments, Australia), and the filter range was 0.3 Hz~1 kHz.

### 5. Stimulus program

#### 5.1 Programmed electrical stimulus

The bipolar electrodes and MAP electrodes were placed on the ventricles to record the MAPs and bipolar ECGs at baseline; 10 min later, PES (8 S1 stimuli plus a single extra-stimulus, S2) was delivered. The S2 was delivered from 125 ms, and reverse scanning was performed at a step of -1 ms. Changes in the AP and bipolar ECG were observed. The ventricular effective refractory periods, types of ventricular arrhythmias, durations, and frequencies were recorded.

#### 5.2 Burst stimulus

The stimulating electrodes were used to deliver burst stimuli at the right ventricular base at a frequency of 50 Hz for 2 s. The burst frequency was 0.2 Hz, and the total stimulation time was less than 3 min [10]. The types of ventricular arrhythmias, durations, and frequencies were recorded. The effective refractory period was defined as the longest S1-S2 interval that failed to result in an excitation wave. The durations of ventricular tachyarrhythmia (VT) were divided into sections of <10 s, 10~30 s, and >30 s. VTs with a duration of >30 s were defined as sustained ventricular tachyarrhythmias.

### 6. Separation of ventricular myocytes

A total of 100 U heparin was administered to the mice via intraperitoneal injection. Ten minutes later, the mice were decapitated, and their hearts were immediately removed and placed in a Langendorff recirculating perfusion apparatus after aortic intubation. Cardiomyocytes were isolated via the step-by-step perfusion method: 1) Tyrode’s-HEPES solution (mM: NaCl 130; KCl 5.4; CaCl_2_ 1.8; MgCl_2_ 1; Na_2_HPO_4_ 0.3; HEPES 10; glucose 10; the pH value was adjusted to 7.4 by adding NaOH) for 5 min; 2) calcium-free Tyrode’s-HEPES solution for 5 min; 3) enzyme-containing digestive fluid (30 ml calcium-free Tyrode’s solution; 0.6 mg/ml collagenase II; 0.1% BSA; 20 mM taurine; 30 μM CaCl_2_) for 15 min; 4) KB solution (mM: taurine 10; glutamic acid 70; KCl 25; KH_2_PO_4_10; glucose 22; EGTA 0.5; the pH value was adjusted to 7.2 by adding KOH) for 5 min. The ventricles were cut off after perfusion. Under a microscope, iris scissors were used to separate the ventricular wall between the left and right ventricles as well as the inner and outer myocardium of the left ventricular free wall [11]. All separation procedures were carried out at room temperature (25°C). The separated cardiomyocytes were placed into KB solution and stored in a refrigerator at 4°C.

### 7. Recording of I-V characteristics of potassium channels in cardiomyocytes

An EPC-9 amplifier was used in the whole-cell patch-clamp technique, and Pulse was used for data recording and analysis. Cells were placed in a perfusion chamber and treated with continuous constant-temperature perfusion of calcium-free Tyrode’s solution at 2 ml/min. The electrode impedance was 2.5–5 MΩ, and intracellular fluid (mM: K-aspartate 110, KCl 20, NaCl 8, MgC1_2_ 1, CaC1_2_ 1, MgATP 4, EGTA 0.1 and 10 HEPES; the pH value was adjusted to 7.2 by adding KOH) was perfused. The series resistance was kept within the range of 4–8 MΩ and at room temperature within the range of 22-25°C. 1) For the I-V characteristics of the total potassium current (I_peak_), the current was derived by the test pulses (500 ms, -40 mV—+60 mV, at a step of 10 mV), the clamping voltage was -80 mV, and the pulse frequency was 0.1 Hz. 2) For recording fast-activated transient outward rectifying potassium current (I_Kur_), the current was derived by first delivering a pre-stimulation (100 ms, -40 mV) to inactivate the transient outward potassium current (I_to,f_) and then by delivering the test pulses (500 ms, -40 mV—+60 mV, at a step of 10 mV). 3) For recording steady-state outward potassium current (I_SS_), the current was derived by adding 100 μM 4-aminopyridine (4-AP) after recording I_Kur_. 4) For recording I_to,f_, the current was derived under the settings of holding potential: -80 mV; testing potential: -40 mV—+60 mV; step: 10 mV; and clamping time: 400 ms. 5) For recording inward rectifying potassium current (I_K1_), the current was derived under the settings of holding potential: -80 mV; testing potential: -120 mV—-40 mV; step: 10 mV; and clamping time: 350 ms.

### 8. Recording of I_to,f_ and I_Ku_ channel kinetics

1) The steady-state inactivation properties of I_to_ and I_Kur_ were obtained by using double-pulse stimuli. The cell membrane potential was kept at -80 mV; extra-stimuli (-110 mV–-10 mV, at a step of 10 mV) were delivered for 1 s, and then, the inactivation properties of I_to_ were recorded under the testing voltage of +30 mV for 1 s. The cell membrane potential was kept at -80 mV; extra-stimuli (-110 mV–-10 mV, at a step of 10 mV) were delivered for 5 s, and then, the pre-stimulation (100 ms, -40 mV) was delivered to inactivate I_to_. The inactivation properties of I_to_ were then recorded under the test pulses (5 s, +30 mV). Standardized ratios (I/I_max_) were calculated by dividing the test current amplitudes under all voltages by the maximum amplitude. Linear fitting of the data was performed using the Boltzmann equation, and inactivation curves of the channels were then obtained. 2) Recovery properties of I_to_ after the time-dependent inactivation: First, the cell membrane potential was kept at -80 mV; clamping pulses (+30 mV, 500 ms) were delivered to inactivate the I_to_ channel; clamping pulses (+30 mV, 500 ms) were delivered at different time intervals, and peak current amplitudes of I_to_ at the time points within 10 ms—500 ms were recorded. Recovery properties of I_Kur_ after the time-dependent inactivation: Immediately after sending the clamping pulses (+30 mV, 1.5 s), pre-stimulation (100 ms, -40 mV) was delivered to inactivate I_to_, and clamping pulses (+30 mV, 500 ms) were delivered at different time intervals (10 ms—3000 ms). Standardized ratios (P2/P1) were calculated by dividing the test current peaks at the time points by the current peaks derived by the inactivation pulses. Exponential curve fitting of the data with respect to the time interval was performed.

### 9. Statistical analysis

Lab chart 7.0 was used to collect and measure the AP graphs. Data analysis was carried out with SPSS16.0. Pulsefit and Origin 8.0 were used for current curve drawing and analysis. The data are expressed in the form of the mean ± standard deviation. Differences with P < 0.05 were considered statistically significant.

## Results

### 1. Assessment of the cardiac hypertrophy model via anatomical parameters and echocardiography

Cardiac ultrasonic examination was performed four weeks after the cardiac hypertrophy model was established, and the results showed that LVEDD, IVSD, LVPWD, and FS in the surgery group were all significantly higher than in the control group. After animals were sacrificed, their hearts were removed and weighed. The results showed that both HW/BW and LVW/BW in the surgery group were significantly higher than in the control group (see Table 1). This finding demonstrated that the cardiac hypertrophy model, induced by thoracic aortic constriction, was successfully established.

**Table 1:** Echocardiographic characteristics of the control group and the surgery group.

	Control group (n = 8)	Surgery group (n = 8)
BW (g)	27.8±0.6	27.7±0.6
HW/BW (mg/g)	4.18±0.11	6.69±0.18*
LVW/BW (mg/g)	4.14±0.10	6.22±0.23*
LVEDD (mm)	3.71±0.04	4.28±0.03*
IVSD (mm)	0.67±0.01	1.36±0.03*
LVPWD (mm)	1.23±0.03	2.22±0.06*
FS (%)	43.0±0.7	25.0±0.4*

BW, body weight; HW, heart weight; LVW, left ventricle weight; LVEDD, left ventricular end diastolic diameter; IVSD, interventricular septal diameter; LVPWD, left ventricle posterior wall diameter; FS, fraction shortening.

*P<0.05 control group vs surgery group.

### 2. Assessment of repolarization phase by surface ECG and MAP

**Fig 1:**
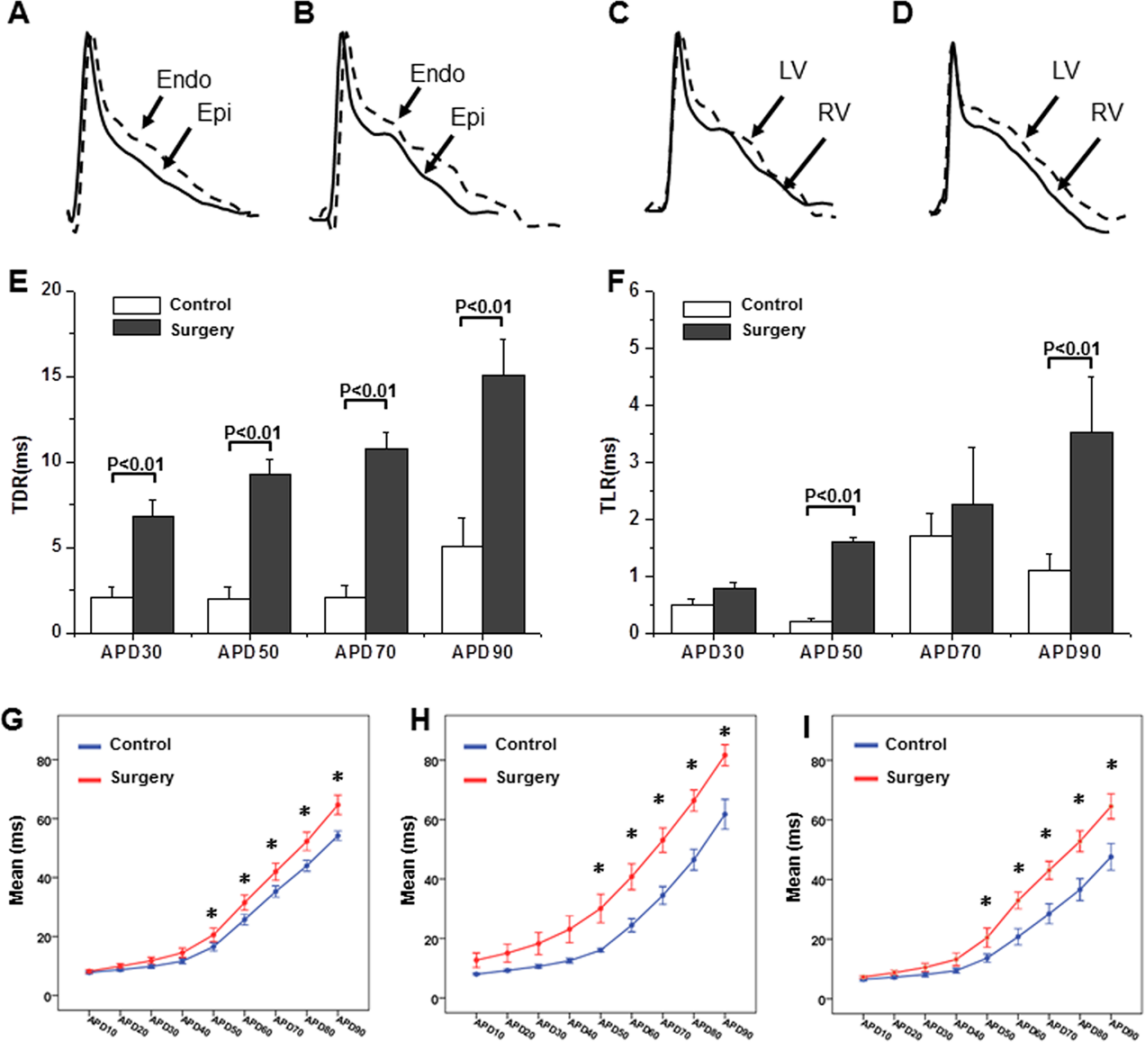
A-B. APD recordings in the endo-myocardium and epi-myocardium in the control (A) and surgery groups (B); C-D. APD recordings in the left and right epi-myocardia in the control (C) and surgery (D) groups; E. The transmural dispersion of repolarization (TDR) in the control and surgery groups; F. Trans-epicardial dispersion of left and right ventricular repolarization (TLR) in the control and surgery groups; G. APD recordings in the endo-myocardium between the control and surgery groups; H. APD recordings in the epi-myocardium between the control and surgery groups; I. APD recordings in the right ventricle between the control and surgery groups.

Analysis of lead II ECGs revealed no significant difference in heart rate between the surgery group and the control group (467.0±29.5 bpm vs. 461.4±60.8 bpm). The surgery group had significantly prolonged QRS intervals, QTc, and JT intervals compared with the control group (QRS: 13.9±3.2 ms vs. 9.9±1.2 ms, P<0.05; QTc: 61.1±9.2 ms vs. 45.4±6.6 ms, P<0.05; JT: 55.9±13.9 ms vs. 42.4±8.8 ms, P<0.05). MAP records showed that, compared to the control group, the surgery group had significantly longer MAP durations in the cardiomyocytes of the epi- and endo-myocardia of the left ventricle and right ventricle at a pacing frequency of 8 Hz. The APD_90_ of two groups at the epi- and endo-myocardia of the left ventricle and right ventricle was 71.7±14.5 ms vs. 51.2±5.8 ms (P<0.01), 84.6±15.3 ms vs. 56.2±6.4 ms (P<0.01), and 67.2±11.9 ms vs. 53.3±8.9 ms (P<0.01), respectively. These changes showed obvious spatial heterogeneity. The transmural dispersion of repolarization (TDR) of APD_90_ between the epi- and endo-myocardia of the left ventricle and the transmural dispersion of repolarization of APD_90_ between the trans-epicardial dispersion of the left and right ventricular repolarization (TLR) were significantly different between the surgery group and the control group (TDR: 15.1±2.1 ms vs. 5.1±1.6 ms, P<0.01; TLR: 3.5 ±1.0 ms vs. 2.1±0.5 ms, P<0.01) (see Fig 1).

During the delivery of the stimuli at S1-S1 cycle lengths of 200 ms, 150 ms, 125 ms, and 100 ms, the ventricular ERPs at different frequencies in the surgery group were all higher than those in the control group: 45.0±12.3 ms vs. 39.5±6.9 ms (P>0.05), 45.4±11.6 ms vs. 39.6±8.3 ms (P>0.05), 47.0±12.1 ms vs. 38.6±7.2 ms (P<0.05), and 44.7±8.9 ms vs. 39.6±8.9 ms (P>0.05), respectively.

### 3. Induction rate of ventricular arrhythmias

**Fig 2:**
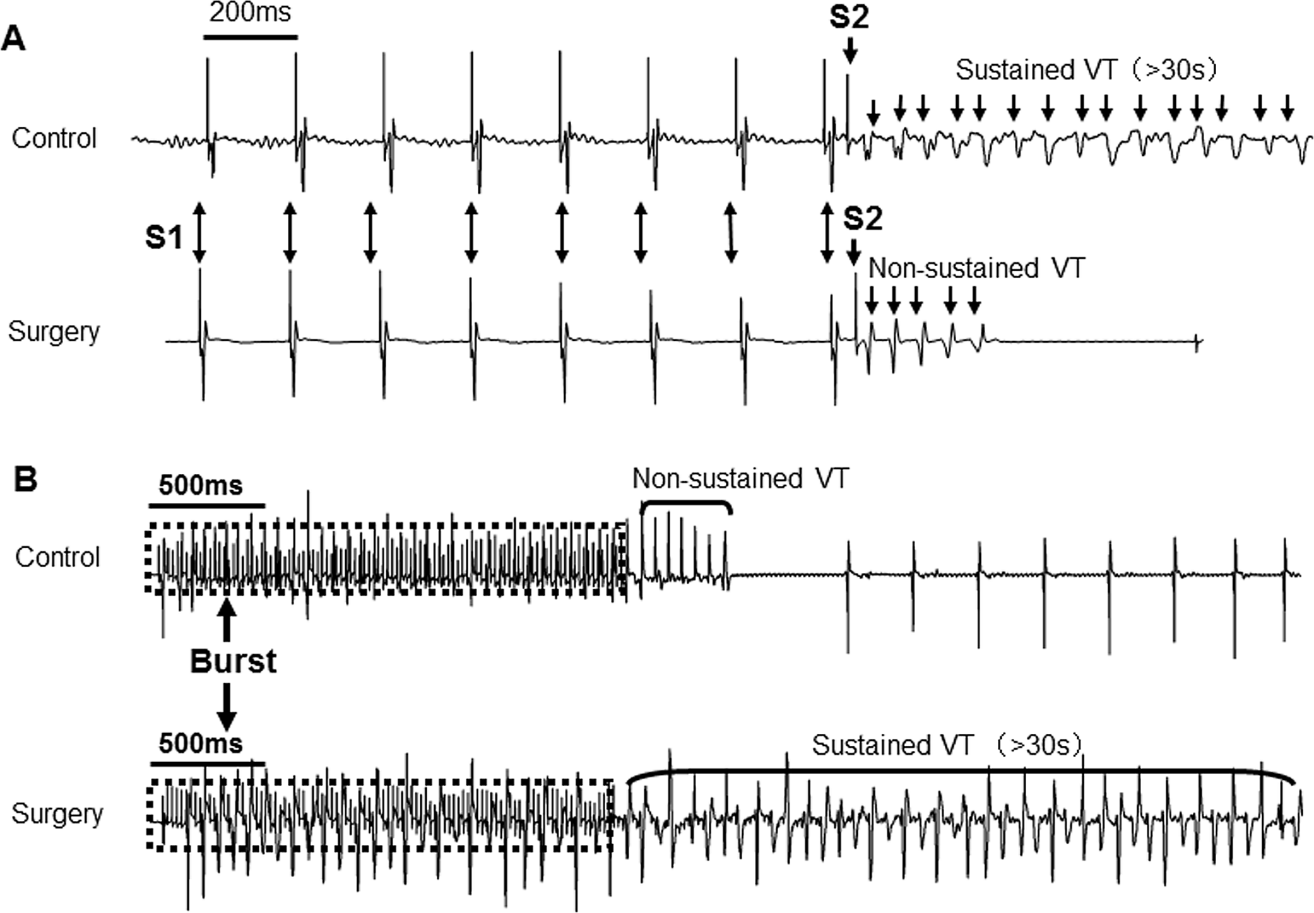
Ventricular tachyarrhythmias were induced under both PES (A) and burst stimuli (B), and the inducibility in the surgery group was higher than that in the control group. Polymorphic ventricular tachyarrhythmias were observed in the surgery group.

Ventricular tachyarrhythmias were induced under both PES and burst stimuli, and both induction rates in the surgery group (41.2% and 23.5%, respectively) were higher than those in the control group (12.5% and 12.5%, respectively). The results also indicated that the hearts of mice in the surgery group were more sensitive to PES. Furthermore, the duration of the arrhythmias in the surgery group was longer than that in the control group. The induction rate of sustained ventricular arrhythmias (>30 s) was 5.9% in the surgery group, and the rate was zero in the control group (See Fig 2). The surgery group had a longer cycle length of arrhythmia waves. In addition, regarding the types of arrhythmias, polymorphic ventricular tachyarrhythmias were observed in the surgery group (5.9%) (See Table 2).

**Table 2:** Incidence of VT induced by PES or burst pacing.

	Control group (n = 16) Surgery group (n = 17)	
Induction rate		
PES	12.5% (2/16)	41.2% (7/17)
Burst stimulus	12.5% (2/16)	23.5% (4/17)
No response	75.0% (12/16)	47.1% (8/17)
Time of duration		
<10 s	12.5% (2/16)	47.1% (8/17)*
10–30 s	0	5.9% (1/17)
>30 s	0	5.9% (1/17)
VT cycle length		
Longest CL (ms)	49.5±10.6	79.4±11.0
Shortest CL (ms)	47.5±9.2	65.6±18.2
VT type		
Mono	12.5%(2/16)	52.9% (9/17)*
Poly	0	5.9% (1/17)

VT, ventricular tachyarrhythmias

*P<0.05 surgery group vs control group.

### 4. Changes in I-V characteristics of potassium currents

**Fig 3:**
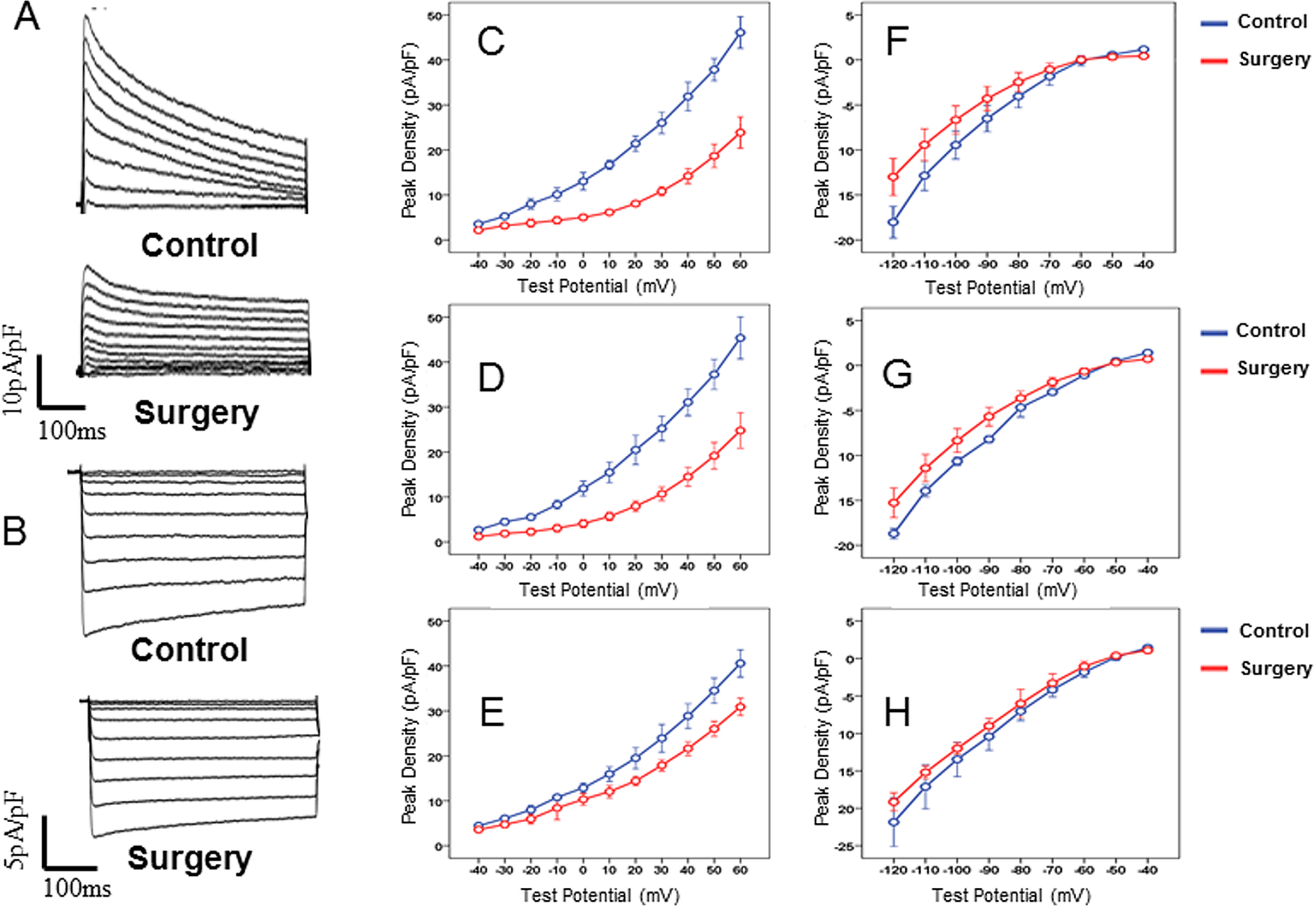
Recording of outward potassium currents (I_Peak_) (A, C, D, E) and inward potassium currents (I_K1_) (B, F, G, H) at different regions, including the epi-myocardium (C, F), endo-myocardium (D, G) and right ventricle (E, H) in both groups.

As revealed by the whole-cell recording of total outward potassium currents (I_Peak_) in cardiomyocytes of different parts (including the endo- and epi-myocardium of the left ventricle and right ventricle), changes in the I_Peak_ density in cardiomyocytes of mice in the surgery group with voltage were significantly lower than those in the control group (see Fig 3). The peak current densities (+60 mV) in the epi-myocardium, endo-myocardium, and right ventricle were 26.6±6.1 pA/pF vs. 48.1±10.3 pA/pF (P<0.05), 21.4±4.7 pA/pF vs. 46.1±8.5 pA/pF (P<0.05), and 30.8±3.8 pA/pF vs. 39.7±5.5 pA/pF (P<0.05), respectively. The total outward potassium currents included many components, and the transient outward potassium current (I_to,f_), ultra-rapid delayed rectifier potassium current (I_Kur_), and steady-state potassium current (I_ss_) played dominant roles. The I-V characteristics of these three outward potassium currents in the surgery group were significantly different from those in the control group, the changes in the endo-myocardium of the left ventricle were particularly significant, and the peak currents of all components (+60 mV) were significantly different compared to the control group (see Table 3). In addition, the potassium current densities in cardiomyocytes of the left ventricle in the surgery group showed obvious transmural heterogeneity; the densities of I_Peak_, I_to,f_, and I_Kur_ in the endo-myocardium of the left ventricle were significantly lower than those in the epi-myocardium of the left ventricle (P < 0.05). However, this heterogeneity was not observed in the control group.

**Table 3:** Peak densities of K^+^ currents in myocytes of epicardium, endocardium and the right ventricle.

	Control group			Surgery group		
	Epi (n = 13)	Endo (n = 10)	RV (n = 11)	Epi (n = 10)	Endo (n = 10)	RV (n = 10)
C_m_ (pF)	152.3±26.5	183.4±29.0*	133.6±22.4	168.4±26.2	207.8±67.6*	199.9±55.0^##^
I_peak_ (pA/pF)	48.1±10.3	46.1±8.5	39.7±5.5	26.6±6.1^##^	21.4±4.7*^##^	30.8±3.8^##^
I_to (_pA/pF)	24.5±8.2	23.6±6.0	26.4±7.2	15.3±3.5^##^	11.3±2.7*^##^	17.4±6.0^#^
I_Kur_ (pA/pF)	21.8±8.6	17.7±4.8	20.4±5.9	14.5±3.9^#^	10.8±2.4*^##^	15.8±4.8
I_ss (_pA/pF)	9.6±1.9	8.1±1.5	9.9±1.3	6.1±1.5^##^	5.3±1.1^##^	7.7±2.3^#^
I_K1_ (pA/pF)	18.0±4.3	18.7±1.3	21.1±4.8	14.3±3.2^#^	15.3±3.6^#^	21.0±7.9

Epi, epicardium; Endo, endocardium; RV, right ventricle

*P<0.05, Endo versus Epi

^#^P<0.05

^##^P<0.01, control group versus surgery group.

The I_K1_ density in cardiomyocytes of the endo- and epi-myocardia of the left ventricle in the surgery group was significantly lower than that in the control group (P<0.05) (see Fig 3); the peak current densities (-120 mV) in the epi-myocardium and endo-myocardium were 14.3±3.2 pA/pF vs. 18.0±4.3 pA/pF (P<0.05) and 15.3±3.6 pA/pF vs. 18.7±1.3 pA/pF (P<0.05), respectively. However, no significant differences between the two groups were found in cardiomyocytes of the right ventricle. In addition, I_K1_ density in the surgery group did not show obvious transmural heterogeneity (see Table 3).

### 5. Changes in the kinetics of I_to_ and I_Kur_ channels

**Fig 4:**
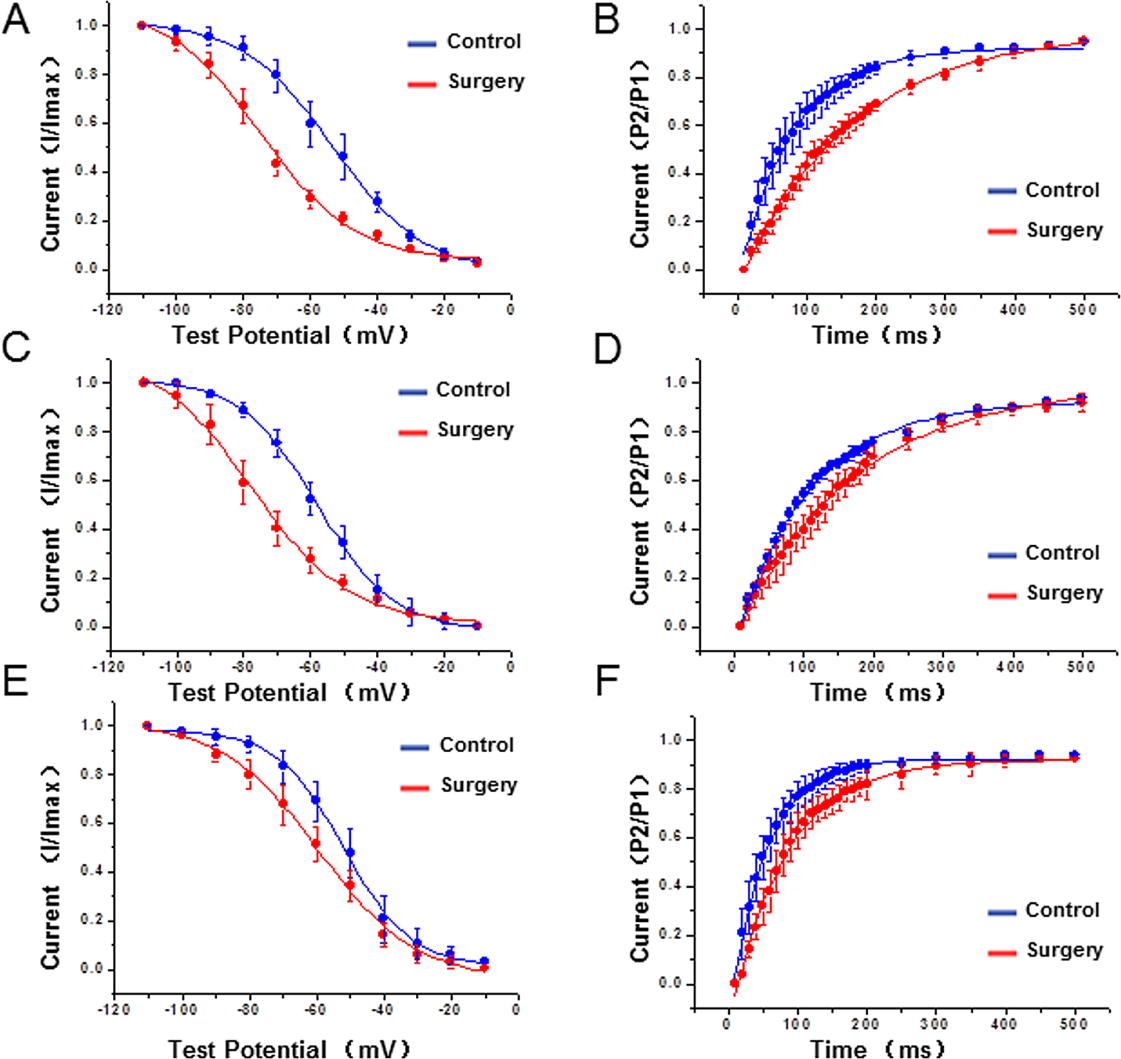
Characteristics of the steady-state inactivation curves (A, C, E) and inactivation recovery curves (B, D, F) of I_to,f_ at different regions, including the epi-myocardium (A, B), endo-myocardium (C, D) and right ventricle (E, F) in both groups.

**Fig 5:**
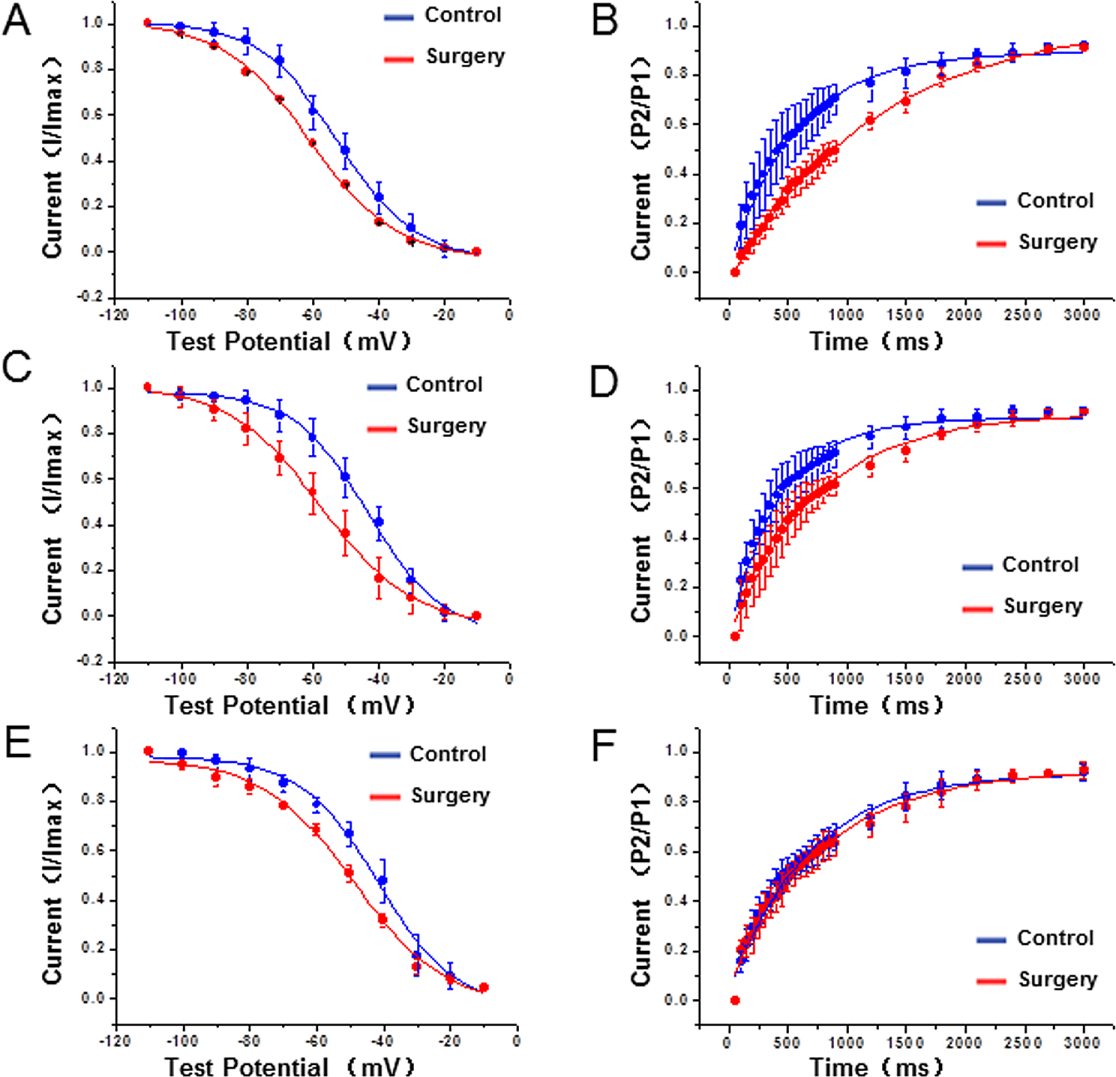
Characteristics of the steady-state inactivation curves (A, C, E) and inactivation recovery curves (B, D, F) of I_Kur_ at different regions, including the epi-myocardium (A, B), endo-myocardium (C, D) and right ventricle (E, F) in both groups.

To further verify whether the decreases in the potassium current densities in cardiac hypertrophy were related to channel kinetics, the kinetic characteristics of I_to,f_ and I_Kur_ were analysed in this study. The results showed that compared to the control group, the steady-state inactivation curves of I_to,f_ in the epi-myocardium, the endo- myocardium, and the right ventricle in the surgery group noticeably shifted to the left (direction of hyperpolarization). However, the inactivation recovery curves of I_to,f_ in the surgery group shifted to the right, and the time constant (τ) was significant longer than in the control group (P<0.01) (Fig 4 and Table 4). In addition, kinetics of I_Kur_ in the surgery group showed the same changing characteristics as I_to,f_. V_1/2_ and k values were greater than in the control group. In addition, the time constant (τ) of I_Kur_ was increased in the epi-myocardium, the endo-myocardium, and the right ventricle compared with those in the control group (Fig 5 and Table 4).

**Table 4:** Kinetics of I_to_ and I_Kur_ channels.

	Control group			Surgeon group		
	Epi (n = 13)	Endo (n = 10)	RV(n = 11)	Epi (n = 10)	Endo (n = 10)	RV (n = 10)
I_to_						
V_1/2_	-53.2±1.1	-53.2±1.1	-51.9±0.8	-74.9±1.3*	-77.8±1.5*	-59.3±1.1*
K	13.6±1.1	11.3±.0.8	9.8±0.7	13.9±1.2	14.7±1.3*	14.3±1.2*
τ	78.6±2.5	108.4±2.8	51.7±1.4	162.1±5.9*	167.2±5.1*	79.7±2.0*
I_Kur_						
V_1/2_	-52.5±0.7	-43.1±0.9	-41.8±1.7	-61.0±0.8*	-57.6±1.0*	-49.1±1.8*
K	11.8±0.7	11.3±0.8	11.6±1.5	13.4±0.8*	14.3±1.1*	13.5±1.8^#^
τ	564.7±24.6	429.6±25.4	659.2±42.3	1246.1±74.2*	732.3±47.1*	767.6±53.5*

Epi, epicardium; Endo, endocardium; RV, right ventricle; τ, time constant

*P<0.01

^#^P<0.05, control group versus surgery group.

## Discussion

Currently, the decrease in Kv density in cardiomyocytes of heart failure patients and animal models has been found to be correlated with a prolonged QT interval and APD [7,12–15]. Previous studies have shown that alterations in I_to_ expression are linked to cardiac hypertrophy [16]. Additionally, the subunits and densities of K^+^ channels, including *I*_to,f_ (Kv4.2 and Kv4.3), *I*_ss_ (Kv2.1), and *I*_kur_ (Kv1.5), contribute to ventricular repolarization [11,17]. However, these previous studies only showed that the pathogenesis of ventricular arrhythmias in cardiac hypertrophy or heart failure is caused by a prolonged QT interval [18]. The present study not only indicated that the extension of the repolarization phase was an important cause of ventricular arrhythmias induced by cardiac hypertrophy but also demonstrated that the repolarizing heterogeneity of cardiac hypertrophy was caused by density and kinetic heterogenous changes in Kv channels. In the present study, the prolonged repolarization was associated with marginally increasing ERP and predominant prolongation of APD_50_-ADP_90_. Thus, the relative refractory period and supranormal period after ERP were prolonged. This change increased the window of vulnerability and facilitated the formation of trigger activity by premature stimuli.

Under normal conditions, K^+^ channels are heterogeneously distributed in the ventricular walls in animals. The subunits of K^+^ channels, including I_to,f_, I_kur_, and Iss, contribute to heterogeneities in ventricular repolarization, and the density of K^+^ currents was more prominent in the epicardium than in the endocardium of mice [11,17]. Consistently, the control group in our study also showed the same profile of heterogeneity in APD and K^+^ channel density between the endo- and epicardium, and this heterogeneity was markedly enhanced in cardiac hypertrophy. Furthermore, kinetic heterogeneity of multiple K^+^ channels were also demonstrated in the present study; these alterations have not been mentioned by previous studies. Notably, the increased dispersion of V_1/2_ and τ of I_to_ and I_Kur_ resulted in heterogeneity of depolarization and repolarization in the ventricles. Thus, the formation of a re-entry mechanism was greatly promoted, and arrhythmia was easily induced by electrical stimulation in cardiac hypertrophy.

Previous studies have indicated that in pressure overload-induced cardiac hypertrophy [14,19,20], the histomorphology of the myocardium is characterized by obvious heterogeneity. Namely, cardiomyocytes in the endo-myocardium of the left ventricle have a higher degree of hypertrophy than cardiomyocytes in the epi-myocardium [14]. This may be because the endo-myocardium has a larger mechanical tension than the epi-myocardium under a high-pressure load. As a result, the increase in the volume of cardiomyocytes in the endo-myocardium is more significant than in the epi-myocardium, and cardiomyocytes in the endo-myocardium have a significantly lower ion channel density. Notably, this study found that cardiomyocytes of the endo-myocardium had more significant APD prolongation, which was associated with the spatial heterogenous decrease in potassium current densities. The changes in the cardiomyocytes of the endo-myocardium were particularly significant and directly influenced the transmural electrophysiological heterogeneity of the ventricular myocardium. Transmural heterogeneity has been found in the hearts of many animals and humans, and the cause of this phenomenon is considered to be the regional differences in the density and type of repolarization K^+^ currents in the myocardium [21]. Several studies have proposed that there is a transmural gradient of I_to_ between the endo-myocardium and epi-myocardium of mice, which is caused by the heterogeneity of expression of the α subunit (Kv4.3) and the calcium-binding auxiliary subunit (KChIP2) [22, 23]. However, whether there are physiologic transmural differences in outward rectifier currents (I_Kur,_ I_Kr_, and I_Ks_) remains controversial. Brunet et al. noted that cardiomyocytes in the epi-myocardium of C57BL6 mice had a higher I_Ks_ density than those in the endo-myocardium. Because the inactivation time constant of I_Ks_ in the endo-myocardium is significantly higher than in the epi-myocardium, they speculated that different types of I_Ks_ channels were distributed on the membrane of cardiomyocytes in the endo- and epi-myocardia. However, the expression levels of Kv1.5 and Kv2.1 fail to indicate a transmural gradient [11]. Marionneau et al. found that in aortic coarctation-induced cardiac hypertrophy, there were no significant differences in the amplitudes of I_to_, I_K,slow_, and I_K1_ between cardiomyocytes in the endo-myocardium and those in the epi-myocardium, and there was no transmural spatial heterogeneity in the expression of channel proteins, I_to_ (Kv4.2, Kv4.3, KChIP2), I_K1_ (Kir2.1, Kir2.2), and I_ss_ (TASK1, TASK2). However, the membrane capacitance (C_m_) increases significantly, thus changing the transmural spatial heterogeneity of potassium currents in the myocardial tissue. This finding indicates that the transmural spatial heterogeneity in cardiac hypertrophy does not depend on changes in potassium channel protein expression and ion currents but depends on heterogeneous changes in the cell volume [14]. The results of our present study also demonstrate that the I_to,f_ and I_Kur_ densities change more significantly in cardiomyocytes of the endo-myocardium and that the changes are consistent with changes in the membrane capacitance.

## Conclusion

The non-uniform attenuation of potassium currents in cardiomyocytes contributes to irregular repolarization in cardiac hypertrophy, and the spatially heterogeneous changes of the channels may directly increase the occurrence of ventricular arrhythmias that accompany cardiac hypertrophy.

## Supporting information

S1 File NC3Rs ARRIVE guidelines checklist. (PDF)
